# Soyasaponins Can Blunt Inflammation by Inhibiting the Reactive Oxygen Species-Mediated Activation of PI3K/Akt/NF-kB Pathway

**DOI:** 10.1371/journal.pone.0107655

**Published:** 2014-09-18

**Authors:** Longying Zha, Jiading Chen, Suxia Sun, Limei Mao, Xinwei Chu, Hong Deng, Junwei Cai, Xuefeng Li, Zhenqi Liu, Wenhong Cao

**Affiliations:** 1 Department of Nutrition and Food Hygiene, School of Public Health and Tropical Medicine, Southern Medical University, Guangzhou, China; 2 Department of Endocrinology, Taihe Hospital, Hubei University of Medicine, Shiyan, China; 3 Department of Medicine, University of Virginia Health System, Charlottesville, Virginia, United States of America; 4 Nutrition Research Institute at Kannapolis, Department of Nutrition, Gillings School of Global Public Health, The University of North Carolina at Chapel Hill, Chapel Hill, North Carolina, United States of America; 5 Department of Medicine (Endocrinology and Metabolism), Duke University School of Medicine, Durham, North Carolina, United States of America; Temple University School of Medicine, United States of America

## Abstract

We and others have recently shown that soyasaponins abundant in soybeans can decrease inflammation by suppressing the nuclear factor kappa B (NF-kB)-mediated inflammation. However, the exact molecular mechanisms by which soyasaponins inhibit the NF-kB pathway have not been established. In this study in macrophages, soyasaponins (A_1_, A_2_ and I) inhibited the lipopolysaccharide (LPS)-induced release of inflammatory marker prostaglandin E_2_ (PGE_2_) to a similar extent as the NF-kB inhibitor (BAY117082). Soyasaponins (A_1_, A_2_ and I) also suppressed the LPS-induced expression of cyclooxygenase 2 (COX-2), another inflammatory marker, in a dose-dependent manner by inhibiting NF-kB activation. In defining the associated mechanisms, we found that soyasaponins (A_1_, A_2_ and I) blunted the LPS-induced IKKα/β phosphorylation, IkB phosphorylation and degradation, and NF-kB p65 phosphorylation and nuclear translocation. In studying the upstream targets of soyasaponins on the NF-kB pathway, we found that soyasaponins (A_1_, A_2_ and I) suppressed the LPS-induced activation of PI3K/Akt similarly as the PI3K inhibitor LY294002, which alone blocked the LPS-induced activation of NF-kB. Additionally, soyasaponins (A_1_, A_2_ and I) reduced the LPS-induced production of reactive oxygen species (ROS) to the same extent as the anti-oxidant N-acetyl-L-cysteine, which alone inhibited the LPS-induced phosphorylation of Akt, IKKα/β, IkBα, and p65, transactivity of NF-kB, PGE_2_ production, and malondialdehyde production. Finally, our results show that soyasaponins (A_1_, A_2_ and I) elevated SOD activity and the GSH/GSSG ratio. Together, these results show that soyasaponins (A_1_, A_2_ and I) can blunt inflammation by inhibiting the ROS-mediated activation of the PI3K/Akt/NF-kB pathway.

## Introduction

Excessive inflammation is associated with all sorts of chronic diseases such as obesity, diabetes, cardiovascular disorders (CVD), alcoholic and non-alcoholic fatty liver, Alzheimer’s disease, some types of cancer, and accelerated aging, etc. [1]–[3]. Importantly, reduction of inflammation by using various phytochemicals from fruits, vegetables, whole grains and other plants may be used to prevent all these chronic diseases [4]–[7]. One example of such phytochemicals is components from soybeans [8], [9].

Consumption of soybean and its products is strongly associated with the reduced prevalence of diabetes, CVDs, Alzheimer’s disease, and cancer and linked to longevity [10], [11]. The associated mechanisms have been under intense investigation but remain to be defined. There is no doubt that phytochemicals from soy products play an important role in all these benefits [12]–[14]. Soybean phytochemicals include isoflavones (0.1–0.3%), phytic acids (1.0–2.2%), phytosterols (0.23–0.46%) and soyasaponins (0.17–6.16%) [13]. Soyasaponins are abundant in soy products and have been classified into 4 groups (A, B, E, and DDMP (2,3-dihydro-2,5dihydroxy -6-methyl-4 H-pyran-4-one)) according to their oleanane-type triterpenoid aglycone structure [15]. Groups A and B are more abundant than the others [15]. Group A (A_1_, A_2_, A_3_, A_4_, A_5_ and A_6_) has soyasapogenol A (SG-A) as its core structural aglycone while group B (I, II, III, IV, and V) contains soyasapogenol B (SG-B) [16]. Interestingly, soyasaponins have been shown to be antimutagenic [17], [18], anticarcinogenic [15], [19], anti-viral [20], [21], hepatoprotective [22], [23], and antioxidant [24], and can reduce plasma level of cholesterol [25], [26]. We have recently shown that members from soyasaponins group A (A_1_ and A_2_) and B (I) can inhibit NO production in the LPS-stimulated macrophages through inhibition of the nuclear factor kappa B (NF-kB)-mediated iNOS expression [27]. Others have also shown that soyasaponins can decrease inflammation by suppressing the NF-kB-mediated transcription of inflammatory genes [28], [29]. Nevertheless, the exact mechanisms by which soyasaponins modulate NF-kB activation have not been defined. In this study, we addressed this issue and found that soyasaponins modulates NF-kB activation through the reactive oxygen species (ROS)-mediated activation of PI3 K/Akt pathway in macrophages.

## Experimental Procedures

### Antibodies and reagents

Soyasaponins with different chemical structures (SS-A_1_, SS-A_2_ and SS-I) were prepared as previously described [27]. Polyclonal antibodies against COX-2 (D5H5), phospho-NF-κB-p65 (Ser^536^), NF-κB-p65, phospho-IκBa (Ser^32^), IkBa, phospho-IKKα/β (Ser^176/180^), IKKβ, phospho-PI3K-p85 (Tyr^458^) and PI3K-p85 were purchased from Cell Signaling Technology, Inc. (Danvers, MA, USA). Antibodies to phospho-Akt (Ser^473^), Akt, lamin B_1_ and β-actin, and lipopolysaccharides (LPS), N-acetyl-L-cysteine (NAC), and 2′, 7′-dichlorofluorescein diacetate (DCF-DA) were from Sigma (Saint Louis, MO, USA). All secondary antibodies used for western blotting were from Rockland Immunochemical, Inc. (Gilbertsville, PA, USA). The pharmaceutical inhibitor of NF-kB (BAY117082) and PI3K (LY294002) were purchased from Santa Cruz Biotechnology, Inc. (Santa Cruz, CA, USA).

### Cell culture

RAW264.7 murine macrophage cells from American Type Culture Collection (ATCC) were cultured in Dulbecco’s Modified Essential Medium (DMEM, Invitrogen) supplemented with 10% (v/v) heat-inactivated fetal bovine serum (FBS, Gibco, Grand Island, NY, USA), 2 mM L-glutamine, and penicillin (100 units/mL)-streptomycin (100 µg/mL) at 37°C in an atmosphere of 5% CO_2_.

### PGE_2_ analysis

Cells were pretreated with SS (SS-A_1_, SS-A_2_ or SS-I) or inhibitors and then stimulated by LPS for 16 h. Prostaglandin E_2_ (PGE_2_) in the culture media was quantified with an enzyme immunoassay (EIA) kit (Cayman Chemical, Ann Arbor, MI, USA, catalog no. 514010).

### Immunoblotting blotting

Following the indicated treatment, cells were rinsed twice with ice-cold PBS and then lysed with a lysis buffer (20 mM Tris-HCl, pH 7.5, 137 mM NaCl, 1 mM Na_2_EDTA, 1 mM EGTA, 1% Triton X-100, 2.5 mM sodium pyrophosphate, 1 mM glycerophosphate, 1 mM Na_3_VO4, 2 µg/mL leupeptin, and 10 µg/mL aprotinin) supplemented with 1 mM phenylmethylsulfonyl fluoride before use. Lysates (30 µg/lane) were resolved in 4–12% Tris glycine gels (Invitrogen) and transferred to nitrocellulose membranes (Bio-Rad). The presence of proteins was detected by immunoblotting with primary antibodies as indicated and alkaline phosphatase- conjugated secondary antisera. Fluorescent bands were visualized using the Odyssey infrared imaging system (ODYSSEY Fc station, LI-COR).

### Real-time fluorescent quantitative polymerase chain reaction (PCR)

Following treatment, cells were harvested and total RNA was isolated using TRIZOL reagent (Sigma) according to the manufacturer’s protocol. First-strand cDNA was synthesized from total RNA using the AMV reverse transcriptase (Promega, Madison, WI, USA) with indicated primers (COX-2: sense 5′-TCTCCAACCTCTCCTACTAC-3′, antisense 5′-GCACGTAGTCTTC GATCACT-3′; β-actin: sense 5′-GATGGTGGGAATGGGTCA GA-3′, antisense 5′-TCCATGTCGTCCCAGTTGGT-3′). PCR was performed in 20 µl volume of mixtures containing 1 µg of cDNA template, 0.5 µM each of the primers, and SYBR Green Real-time PCR Master Mix (TOYOBO, Kita-ku, Osaka, Japan, code no. QPK-201 & 201 T) by using a continuous fluorescence detector (ABI 7500, USA). Samples were normalized by dividing the quantity of the COX-2 gene by the value of a house-keeping gene (β-actin) in the same sample. Results were presented as COX-2 mRNA relative expression (folds to the negative control which denoted as 1).

### Preparation of cytoplasmic and nuclear extracts

Cells were washed with ice-cold PBS twice, scraped, collected and transferred into clean centrifuge tubes. Cell pellets were processed to separate the cytoplasmic and nuclear fractions using the CHEMICON’s nuclear extraction kit (EMD Millipore Corporation, Temecular, CA, USA, catalog no. 2900) according to the manufacturer’s instructions. Protein concentration in the supernatant was quantified by Pierce Bicinchoninic Acid (BCA) protein assay kit (Thermo Scientific, Rockford, IL, USA, catalog no. 23225 & 23227).

### pNF-kB-Luc reporter gene assay

The commercially available plasmid pNF-kB-Luc (Stratagene, Jalla, CA, USA) was used to assess the NF-kB activity. Briefly, RAW264.7 cells were cultured in a 60-mm dish. After confluence, cells were then transfected with the plasmid by using Lipofect-AMINE reagent (Life Technologies, Inc. Grand Island, NY). After 24 h of incubation, cells were trypsinized and reseeded equally into 6-well plates. After another 24 h, cells were treated as noted. Cells were subsequently harvested and luciferase activities were determined according to manufacturer’s instructions. Results were described as relative luciferase activity (fold difference compared to the negative control).

### Detection of reactive oxygen species (ROS)

Fluorescent DCF-DA was used to detect intracellular ROS. The nonfluorescent dye can freely permeate into cells, in which it is deesterified to form the ionized free acid (dichlorofluorescein) and reacts with ROS to form fluorescent 2′, 7′-dichlorofluorescein (DCF). Following indicated treatment, cells were washed with PBS three times and loaded with 20 µmol/L DCF-DA at 37°C for 30 min to allow complete deesterification of the dye. DCF fluorescence was quantified by using a fluorescence plate reader (Spectramax Gemini EM, Molecular Devices, Eugene, OR) at the excitation and emission wavelengths of 490 nm and 530 nm, respectively.

### Determination of SOD activity, GSH/GSSG ratio and malondialdehyde (MDA) levels

Commercial assay kits were correspondingly used for the determination of superoxide dismutase (SOD determination kit, Sigma-Aldrich, Saint Louis, MO, catalog no. 19160), reduced and oxidized glutathione (BIOXYTECH GSH/GSSG-412, OxisResearch, Manhattan Beach, CA, catalog no. 21040), and malondialdehyde (TBARS Assay kit, Cayman Chemical Company, Ann Arbor, MI, catalog no. 10009055) according to the recommended protocols.

### Statistical analysis

Statistical analyses were performed using one way analysis of variance (one-way ANOVA) and LSD multiple comparison tests by SPSS 11.5 statistical software (SPSS Inc., Chicago, IL, USA). Data are presented as means ± SD of three independent experiments. Significant values (*P*<0.05) were marked with an asterisk (*) or an octothorpe (#).

## Results

### Soyasaponins blunt the LPS-induced release of inflammatory marker PGE_2_ in macrophages

**Figure 1:**
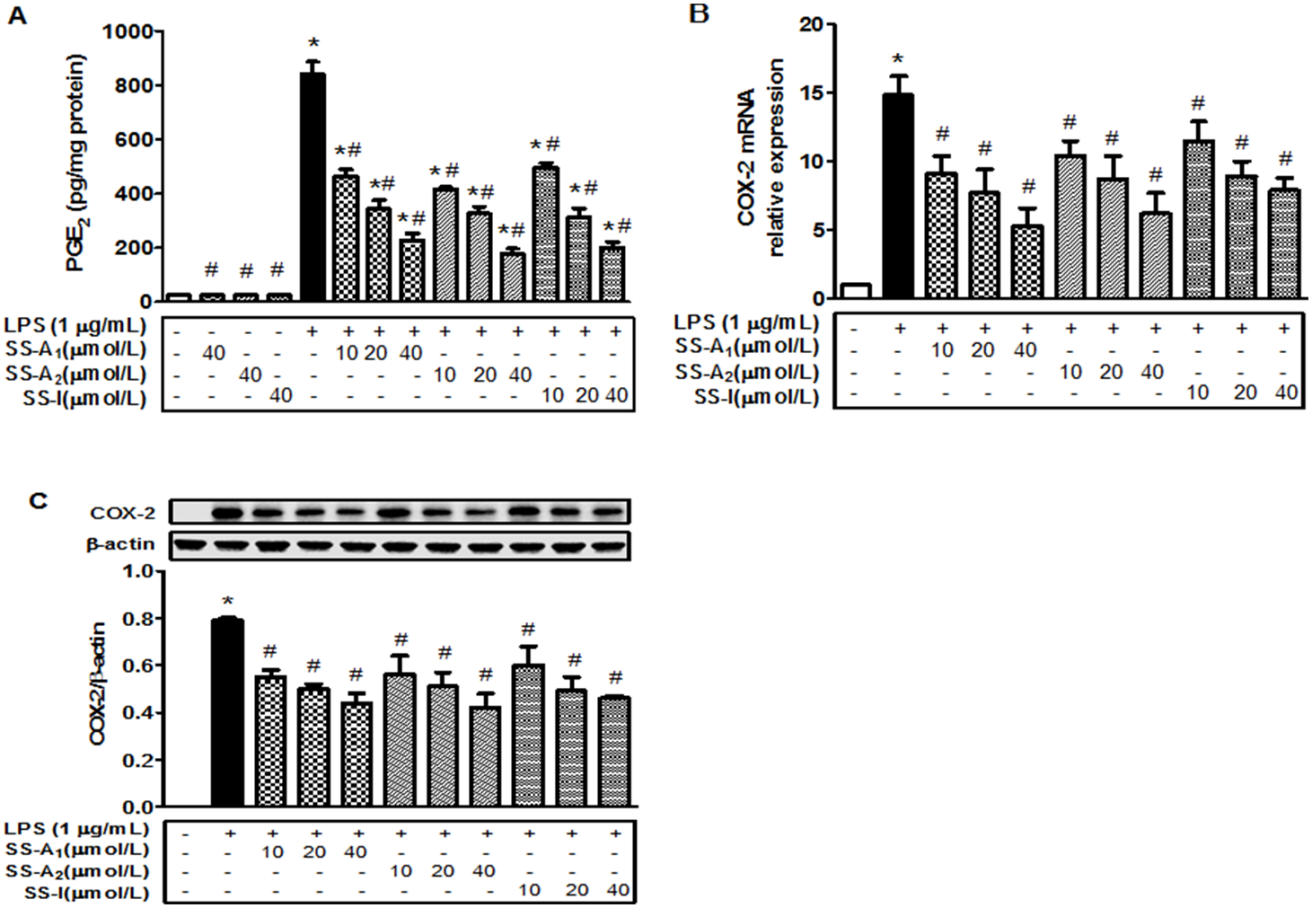
Soyasaponins blunt the LPS-induced release of inflammatory markers in macrophages. Cells were pre-treated with increasing amount of soyasaponins (A_1_, A_2_ and I) for 30 min and then treated with LPS (1 µg/mL) for 16 h as noted. Some cells were treated with soyasaponins alone for the same amount of time. PGE_2_ in the media was then quantified with enzyme immunoassays (**A**). Total RNA was isolated and COX-2 mRNA level was determined by using real-time PCR (**B**). Protein level of the COX-2 gene was evaluated with immunoblotting (**C**). Results represent means ± SD of 3 independent experiments. *: *P*<0.05 vs. control. #: *P*<0.05 vs. LPS alone.

To determine the effect of soyasaponins on inflammation, RAW264.7 macrophages were treated with LPS in the absence or presence of an increasing amount of soyasaponins, followed by measurements of inflammatory markers (PGE_2_ and COX-2). As shown in **Fig. 1A**, the level of PGE_2_ in the control was very low, and incubation with soyasaponins (A_1_, A_2_, or I) alone at 40 µM for 16 h did not alter the level of PGE_2_. PGE_2_ level was dramatically increased by LPS, but the elevation was prevented by all soyasaponins (A_1_, A_2_ and I) in a concentration-dependent manner. Similarly, expression of the COX-2 gene (mRNA and protein) was stimulated by LPS (**Fig. 1B–C**), and the stimulation was blunted by all soyasaponins (A_1_, A_2_ and I) in a concentration-dependent manner. Treatment of macrophages with soyasaponins at all these concentrations did not cause detectable cytotoxicity (data not shown). Together, these results show that soyasaponins can prevent or reduce the level of inflammation.

### Soyasaponins blunt the release of inflammatory marker PGE_2_ in macrophages similarly as an inhibitor of NF-kB

**Figure 2:**
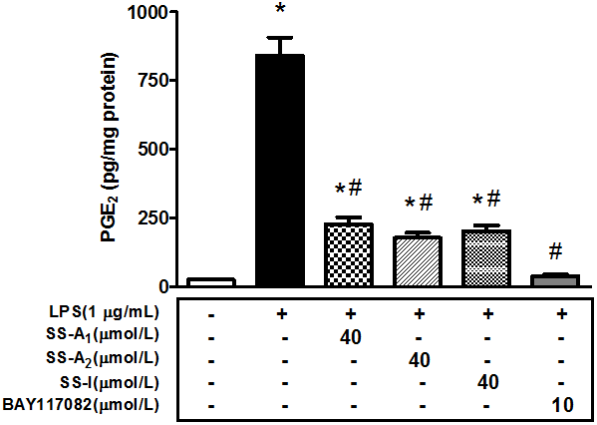
Soyasaponins blunt the release of inflammatory marker PGE_2_ in macrophages similarly as an inhibitor of NF-kB. Cells were pre-treated with the NF-kB inhibitor (BAY117082, 10 µmol/L) or soyasaponins (A_1_, A_2_ and I, 40 µmol/L) for 30 min and then stimulated with LPS (1 µg/mL) for 16 h as indicated. PGE_2_ in the media was quantified with enzyme immunoassays. Results are presented as means ± SD of 3 independent experiments. *: *P*<0.05 vs. control. #: *P*<0.05 vs. LPS alone.

The NF-kB-mediated signaling pathway is the primary player in the LPS-induced inflammatory responses [30]. To determine the mechanism by which soyasaponins blunt the LPS-stimulated inflammation, macrophages were pretreated with soyasaponins (A_1_, A_2_, or I) or the specific inhibitor of NF-kB, BAY117082, prior to the LPS stimulation. The level of PGE_2_ in the media was subsequently quantified. As shown in **Fig. 2**, soyasaponins (A_1_, A_2_ and I) blunted the LPS-induced release of PGE_2_ in the same way as the NF-kB inhibitor BAY117082. These results imply that the effect of soyasaponins on the LPS-stimulated PGE_2_ release is associated with inhibition of the NF-kB signaling pathway.

### Soyasaponins inhibit the LPS-induced activation of NF-kB in macrophages

**Figure 3:**
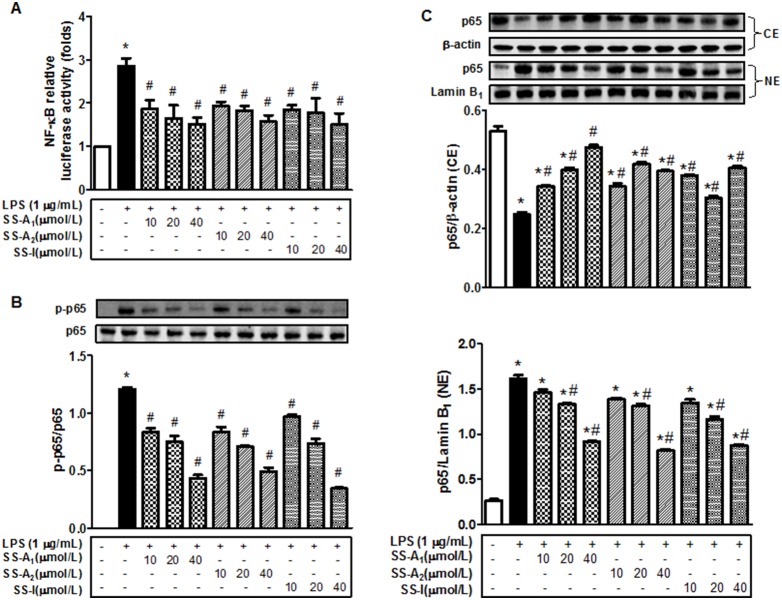
Soyasaponins inhibit the LPS-induced activation of NF-kB in macrophages. (**A**) The pNF-kB-Luc reporter plasmid was introduced into macrophages via transient transfection and then stimulated for 16 h with 1 µg/mL LPS in the absence or presence of increased concentrations of soyasaponins (10–40 µmol/L). The level of luciferase activity was quantified determined and presented as fold of the negative control, which was set as 1. (**B**) Macrophages were pre-treated with 10, 20, or 40 µmol/L of soyasaponins (A1, A2 and I) for 2 h, and then stimulated with LPS (1 µg/mL) for 15 min as noted. Levels of targets proteins in the whole cell lysates were evaluated with immunoblotting. (**C**) Macrophages were pre-treated with 10, 20, or 40 µmol/L of soyasaponins (A_1_, A_2_ and I) for 2 h, and then stimulated with LPS (1 µg/mL) for 1 h as noted. The level of p65 in the nuclear and cytosolic extracts was determined by using immunoblotting. Lamin B_1_ was used as the nuclear protein marker and β-actin as the cytosolic protein marker. Results are presented as means ± SD of 3 independent experiments. *: *P*<0.05 vs. control. #: *P*<0.5 vs. LPS alone.

To define the involvement of the NF-kB pathway in the soyasaponins-mediated suppression of the LPS-induced inflammation, activity of the NF-kB signaling pathway was evaluated by measuring the level of a luciferase-labeled NF-kB. As shown in **Fig. 3A**, the LPS-induced activation of NF-kB was blunted by all soyasaponins (A_1_, A_2_, and I). LPS stimulated NF-kB p65 phosphorylation and the stimulation was blunted by using soyasaponins A_1_, A_2_, or I (**Fig. 3B**). Moreover, LPS enhanced the nuclear translocation of the NF-kB p65 subunit, and the enhancement was prevented largely by soyasaponins A_1_, A_2_, or I (**Fig. 3C**). Together, these results demonstrate that soyasaponins can effectively inhibit the whole pathway of the NF-kB signaling.

### Soyasaponins influence NF-kB activation by modulating phosphorylation and degradation of IkB

**Figure 4:**
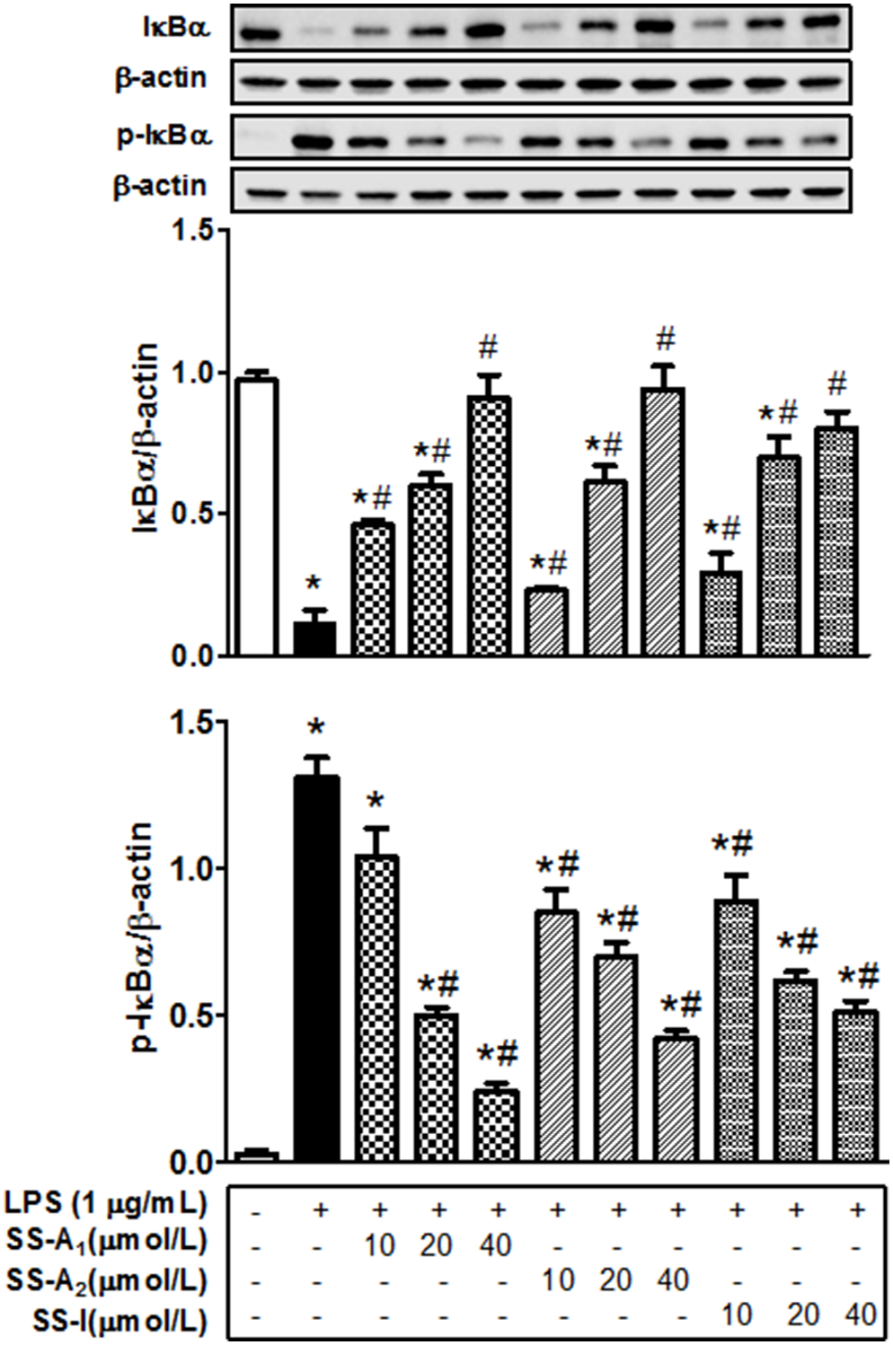
Soyasaponins influence the LPS-stimulated activation of NF-kB through modulating the degradation and phosphorylation of IkBα in macrophages. Macrophages were pre-treated with 10, 20, or 40 µmol/L of soyasaponins (A_1_, A_2_ and I) for 2 h, and then stimulated with LPS (1 µg/mL) for 10 min or 30 min as indicated. Phosphorylation and degradation of IkBα were detected by immunoblotting, and β-actin was used as a control. Results are presented as means ± SD of 3 independent experiments. *: *P*<0.05 vs. control. #: *P*<0.05 vs. LPS alone.

Since phosphorylation and degradation of IkB protein are critical steps for activation of the NF-kB signaling pathway [31], we measured the effect of soyasaponins on the LPS-initiated phosphorylation and degradation of NF-kB. As shown in **Fig. 4**, LPS stimulated the phosphorylation and degradation of IkB, and the stimulation was prevented largely by all soyasaponins (A_1_, A_2_, or I). These results support the notion that soyasaponins influence NF-kB activation by modulating phosphorylation and degradation of IkB.

### Soyasaponins influence NF-kB activation through modulation of IKKα/β phosphorylation

**Figure 5:**
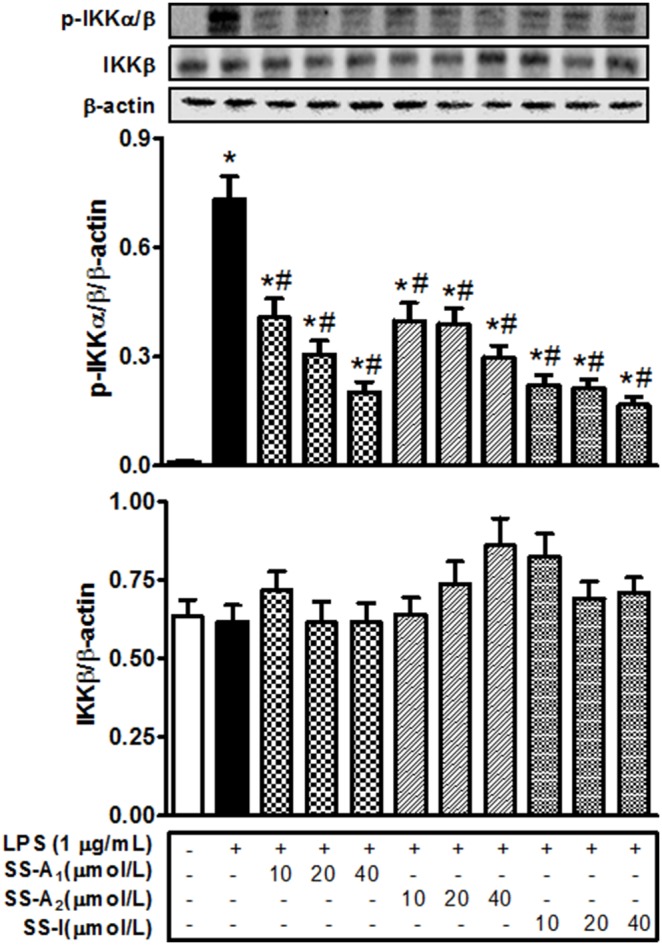
Soyasaponins influence the LPS-stimulated activation of NF-kB through modulation of IKKα/β phosphorylation in macrophages. Macrophages were pre-treated with 10, 20, or 40 µmol/L of soyasaponins (A_1_, A_2_ and I) for 2 h, and then stimulated with LPS (1 µg/mL) for 15 min as indicated. Phospho-IKKα/β and total IKKβ were detected by immunoblotting and β-actin was used as a control. Results are presented as means ± SD of 3 independent experiments. *: *P*<0.05 vs. control. #: *P*<0.05 vs. LPS alone.

To further investigate the mechanism of soyasaponin suppression of NF-kB activation, we examined the effect of soyasaponins (A_1_, A_2_ and I) on the LPS-stimulated phosphorylation of IKKα/β, an upstream regulator of the phosphorylation and degradation of IkB [32]. As shown in **Fig. 5**, LPS stimulated phosphorylation of IKKα/β and the stimulation was blunted by soyasaponin A_1_, A_2_, or I. These results further support the notion that soyasaponins can inhibit activation of NF-kB pathway via the classical cascade of the signaling pathway.

### Soyasaponins inhibit the LPS-induced PI3K/Akt activation in macrophages

**Figure 6:**
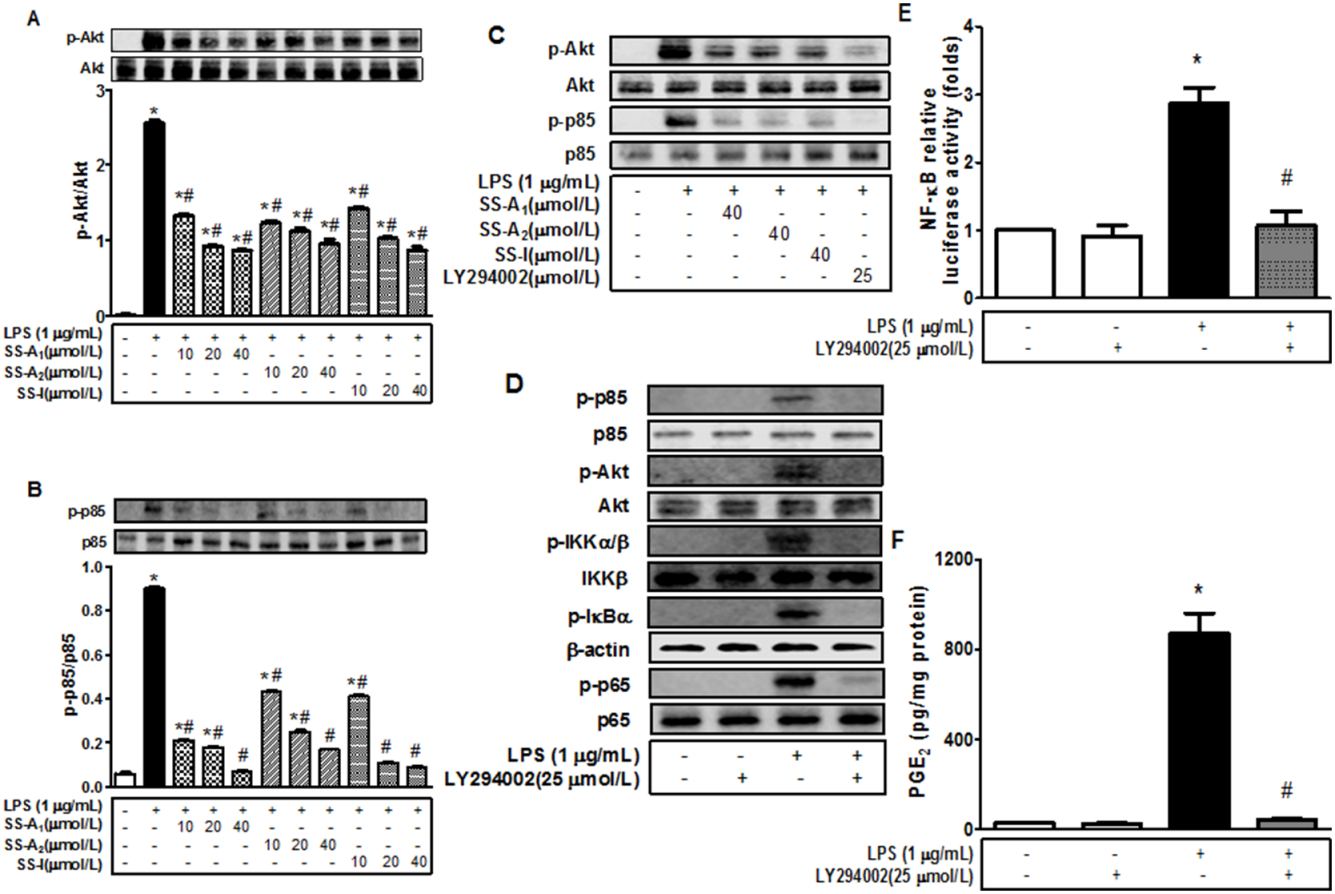
Soyasaponins prevent the LPS-induced PI3K/Akt activation in macrophages. Macrophages were pre-treated with 10, 20, or 40 µmol/L of soyasaponins (A_1_, A_2_ and I) for 2 h, and then stimulated with LPS (1 µg/mL) for 30 min as noted. Phospho- and total Akt and p85 were detected by using immunoblotting (**A–B**). (**C**) Macrophages were pre-treated with 40 µmol/L of soyasaponins (A_1_, A_2_ or I) or 25 µmol/L of LY294002 (a PI3K inhibitor) for 2 h, and then stimulated with LPS (1 µg/mL) for 30 min as noted. Phospho- and total of Akt and p85 were subsequently detected by using immunoblotting. (**D**) Macrophages were pre-treated with 25 µmol/L of LY294002 for 2 h and then stimulated with LPS (1 µg/mL) for 30 min, 15 min or 10 min as indicated. Phosph- and/or total p85, Akt, IKKα/β, IkBα and p65 were detected by using immunoblotting. (**E**) The pNF-kB-Luc plasmids were introduced into macrophages using transient transfection for overnight, and then stimulated with 1 µg/mL of LPS in the absence or presence of LY294002 (25 µmol/L) for 16 h as noted. The level of luciferase activity was evaluated and presented as fold of the negative control. (**F**) Macrophages were pre-treated with 25 µmol/L of LY294002 for 2 h, and then stimulated with 1 µg/mL of LPS for 16 h, followed by measurement of PGE_2_ in the media with an enzyme immunoassay. Results are presented as means ± SD of 3 independent experiments. *: *P*<0.05 vs. control. #: *P*<0.05 vs. LPS alone.

It has been shown that LPS can activate the PI3 K/Akt pathway, which in turn has been shown to be able to activate the NF-kB signaling pathway through IKKα/β in other cell types [33], [34]. To determine whether or not soyasaponins blunted the LPS-induced activation of NF-kB via this pathway in macrophages, we examined the effect of soyasaponins on the LPS-induced phosphorylation of Akt and p85 (a regulatory subunit of PI3 K). Indeed, LPS induced marked phosphorylation of both Akt and p85 in macrophages (**Fig. 6A–B**), and the induction was blunted by soyasaponin (A_1_, A_2_ and I) in a dose-dependent manner. Interestingly, inhibition of PI3 K with LY294002 completely abolished the LPS-induced activation of p85 and Akt (**Fig. 6C**) and the whole cascade of the NF-kB signaling components including phosphorylations of p85, Akt, IKKα/β, IkBα and p65, and NF-kB activity (**Fig. 6D**). Besides, inhibition of PI3 K with LY294002 markedly decreased the NF-kB-dependent luciferase expression induced by LPS (**Fig. 6E**). It is noteworthy that the PI3 K inhibitor LY294002 dramatically reduced the LPS-induced release of PGE_2_ (**Fig. 6F**) similarly as soyasaponins (**see** **Fig. 1**). Together, these results imply that the PI3 K/Akt pathway mediates NF-kB activation and soyasaponins can blunt the NF-kB-mediated inflammation probably by inhibiting the PI3 K/Akt pathway in macrophages.

### Soyasaponins blunts the LPS-induced ROS production in macrophages

**Figure 7:**
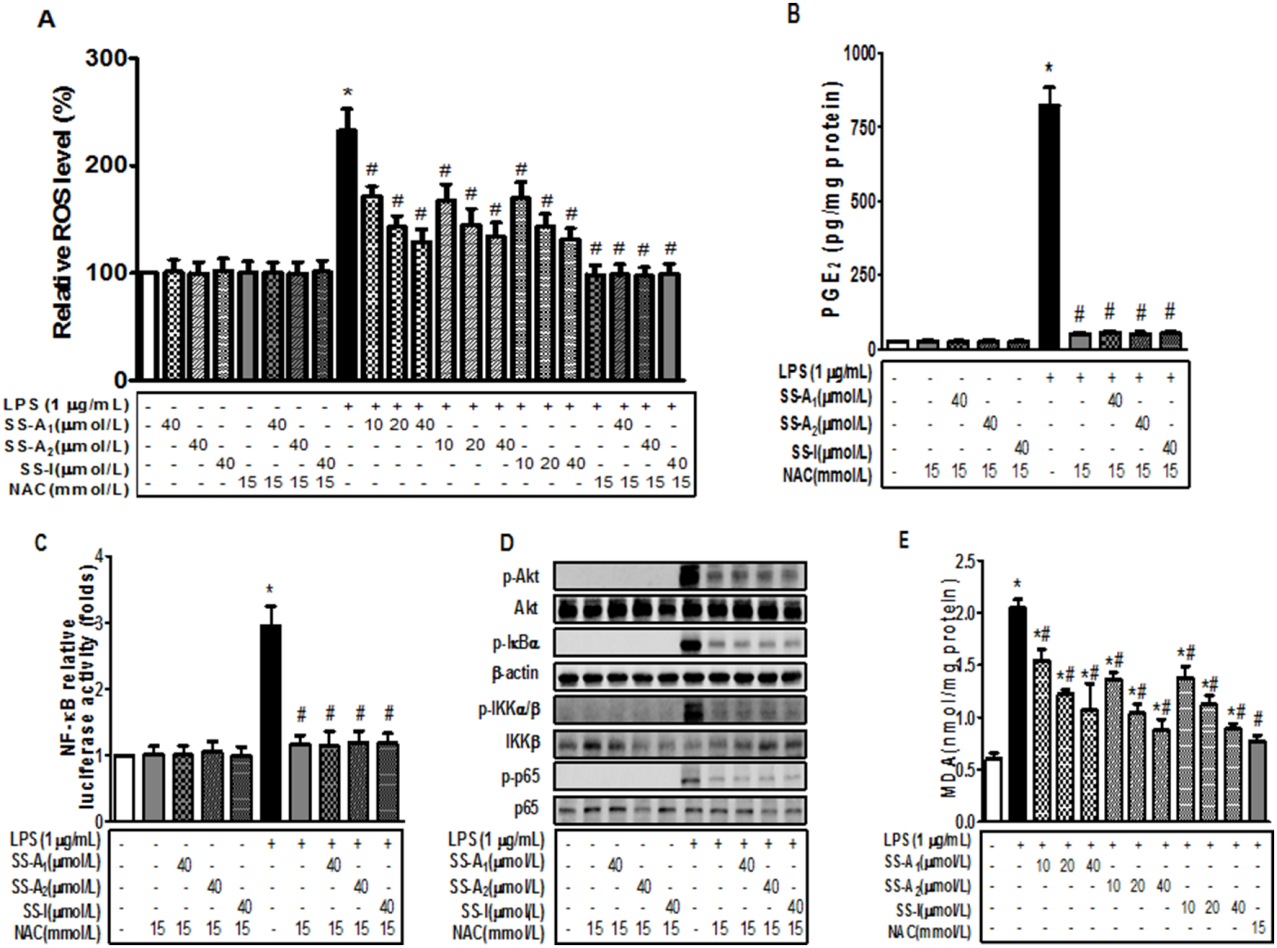
Soyasaponins blunt the LPS-induced ROS production in macrophages. Macrophages were pre-treated with NAC (15 mmol/L) or/and soyasaponins (A_1_, A_2_ or I) for 30 min, and then stimulated with LPS (1 µg/mL) for 16 h as noted. Intracellular ROS level was subsequently quantified by using fluorescent DCF-DA probe and quantified as described in *Materials and Methods* (**A**). The level of PGE_2_ in the media was determined by using an enzyme immunoassay (**B**). (**C**) The pNF-kB-Luc reporter plasmid was introduced into macrophages via transient transfection for overnight, and then stimulated for 16 h with 1 µg/mL LPS in the presence of NAC or NAC plus 40 µmol/L of SS (A_1_, A_2_ or I) as noted. The level of luciferase activity was determined and presented as the fold of the negative control. (**D**) Macrophages were pre-treated with NAC alone or NAC plus 40 µmol/L of SS (A_1_, A_2_ or I) for 2 h, and then stimulated with LPS (1 µg/mL) for 10 min (for detection of p-IkBα and β-actin), 15 min (for detection of p-IKKα/β, IKKβ, p-p65 and p65) or 30 min (for detection of p-Akt and Akt) as noted. Protein levels were detected by using immunoblotting. β-actin was used as a loading control. (**E**) Macrophages were pre-treated with NAC (15 mmol/L) or soyasaponin (A_1_, A_2_ or I) for 30 min, and then stimulated with LPS (1 µg/mL) for 16 h as noted, followed by measurements of MDA in the whole cell lysates. Results are presented as means ± SD of 3 independent experiments. *: *P*<0.05 vs control. #: *P*<0.05 vs LPS alone.

It has been shown that ROS can mediate the LPS-induced inflammation through the Akt-mediated activation of NF-kB in hepatocytes [35]. To determine whether this is also true in macrophages and the targets of soyasaponins, we investigated the potential role of ROS. As shown in **Fig. 7A**, treatment with LPS elevated the intracellular level of ROS, but the elevation was prevented by soyasaponin A_1_, A_2_ or I in a dose-dependent manner. Treatment with soyasaponin (A_1_, A_2_ or I) alone did not affect ROS production. When a well-established anti-oxidant, NAC, was added, the LPS-induced ROS production was completely blocked (**Fig. 7A**). The co-treatment with NAC and soyasaponins (A_1_, A_2_ or I) did not further reduce ROS production, indicating that soyasaponins suppressed ROS to the same extent as NAC. Furthermore, treatment with NAC abolished the LPS-induced production of PGE_2_ (**Fig. 7B**), NF-kB activation (**Fig. 7C**), and phosphorylation of Akt, IkBα, IKKα/β and p65 (**Fig. 7D**). Co-treatment with NAC and soyasaponins did not further augment the inhibitory ability of NAC (**Fig. 7B–D**). Finally, the LPS-induced production of oxidized lipid malondialdehyde (MDA) was also prevented by NAC and soyasaponins to the same extent (**Fig. 7E**). Taken together, these results suggest that soyasaponins (A_1_, A_2_ or I) can blunt the LPS-induced inflammation in macrophages by suppressing the ROS-mediated PI3 K/Akt and NF-kB activation.

### Soyasaponins increase SOD activity and GSH/GSSG ratio

**Figure 8:**
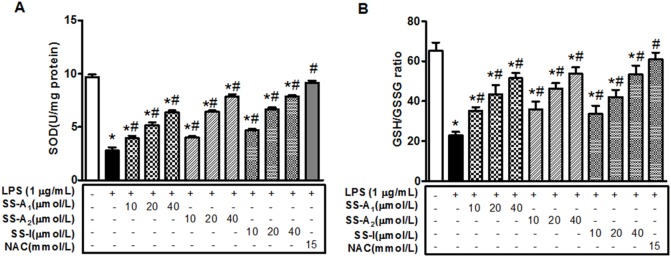
Soyasaponins increase SOD activity and GSH/GSSG ratio in the LPS-stimulated macrophages. Macrophages were pre-treated with NAC (15 mmol/L) or soyasaponin (A_1_, A_2_ or I) for 30 min, and then stimulated with LPS (1 µg/mL) for 16 h as noted, followed by measurements of SOD activity (**A**) and GSH/GSSG ratio (**B**). Results are presented as means ± SD of 3 independent experiments. *: *P*<0.05 vs. control. #: *P*<0.05 vs. LPS alone.

It is known that the immune cells use ROS to support their functions and on the other hand they also need adequate amounts of antioxidation defense to avoid the adverse effect of excessive ROS [36]. Therefore, we further investigated the effects of soyasaponins (A_1_, A_2_ or I) on SOD activity and GSH/GSSG ratio in the LPS-stimulated macrophages. As shown in **Fig. 8**, LPS markedly reduced SOD activity and the GSH/GSSG ratio, whereas treatment of NAC completely abolished this effect. Similarly, soyasaponins (A_1_, A_2_ or I) reversed the suppressive effect of LPS on SOD activity and GSH/GSSG ratio in a dose-dependent manner. Together, these results show that soyasaponins promote the anti-oxidation system.

## Discussion

We and others have recently discovered that soyasaponins can antagonize inflammation and inhibit the NF-kB signaling pathway. Here we further defined the exact effect of soyasaponins on each component of the NF-kB signaling cascade and made some new observations, in particular, about the initial target of soyasaponin modulation of the NF-kB signaling pathway.

First, soyasaponins modulates the NF-kB signaling likely by inhibiting the PI3K/Akt-mediated signaling. It has previously been shown that the PI3K/Akt signaling pathway can be an upstream activator of the NF-kB signaling cascade [33], [34]. In this study, our results clearly show that soyasaponins can modulate every step of the NF-kB signaling pathway similarly as the specific inhibitor of NF-kB. In exploring how soyasaponins influence the initiation steps of NF-kB pathway, we found that soyasaponins could reduce the LPS-induced activations of both PI3K/Akt and NF-kB signaling pathways to the same extent as either the PI3K or NF-kB inhibitor. This implies that soyasaponins modulates the NF-kB signaling through the PI3K/Akt signaling pathway. This observation is important not only for understanding how soyasaponins can modulate the NF-kB signaling and inflammation directly, but also for fully understanding the comprehensive effects of soyasaponins on metabolism because the PI3K/Akt-dependent signaling is the central regulator of the insulin-mediated metabolism [37]. We have recently shown that the basal level of the PI3K/Akt-dependent signaling is increased in the diet-induced insulin resistance and hyperinsulinemia [38]. Insulin resistance/hyperinsulinemia is a precursor and key component of all sorts of metabolic diseases such as metabolic syndrome, type 2 diabetes mellitus, atherosclerotic heart and brain disorders, fatty liver, Alzheimer’s disease, some types of cancer, and aging [39]–[47]. Therefore, soyasaponins can be potentially used to prevent and/or reverse all these major health problems by neutralizing the excessive basal activity of PI3K/Akt signaling. In support of this notion, various reports have shown that consumption of soybeans or their products is associated with a lower rate of cancer [17], [48], [49]. The suppressive effect of soyasaponins on the PI3K/Akt pathway discovered in this study might be an important contributor to the reduced rate of cancer.

Another important finding from this study is that soyasaponins can modulate the NF-kB signaling by scavenging ROS through up-regulation of the most important anti-oxidation system SOD. Specifically, treatment with soyasaponins decreased the LPS-elevated levels of ROS and MDA while increasing the activity of SOD. Typically, SOD activity is increased in the presence of the elevated production of ROS as a defensive mechanism against oxidative stress [50]. In this study, the presence of soyasaponins led to decreased ROS level together with increased SOD activity. That strongly indicates that soyasaponins can directly promote the SOD-mediated anti-oxidation system. Previously, soyasaponins have been shown to have antioxidant activities but the conclusion is debatable. Specifically, Yoshiki and Okubo reported that 1 mg/mL of DDMP soyasaponin (βg) scavenged superoxide at a degree equivalent to 17.1 units of SOD/mL [51]. Another study showed that soyasaponin I could scavenge DPPH radicals with a 50% inhibitory concentration of 70.2 µM comparable to the DPPH radical scavenging activity of α-tocopherol (IC_50_ = 52.1 µM) [29]. However, others have shown that soyasaponin I (βg) did not have scavenging activity due to lack of DDMP moiety [51]. Here we show that soyasaponins scavenge ROS by activating SOD although the exact mechanism remains to be defined. It is well known that ROS can activate all sorts of intracellular signaling pathways such as MAPK (p38, JNK, and ERK1/2), PKCs, NF-kB, and PI3K/Akt etc. [52]. Since soyasaponins can reduce ROS production likely through promoting SOD activity, it is likely that soyasaponins can also modulate these signaling pathways and then influence many other aspects of cellular functions. Defining the effects of soyasaponins in all these pathways and their impact on various cellular functions and activities will definitely help explain the wide spectrum of beneficial effects of soyasaponins on human health.

**Figure 9:**
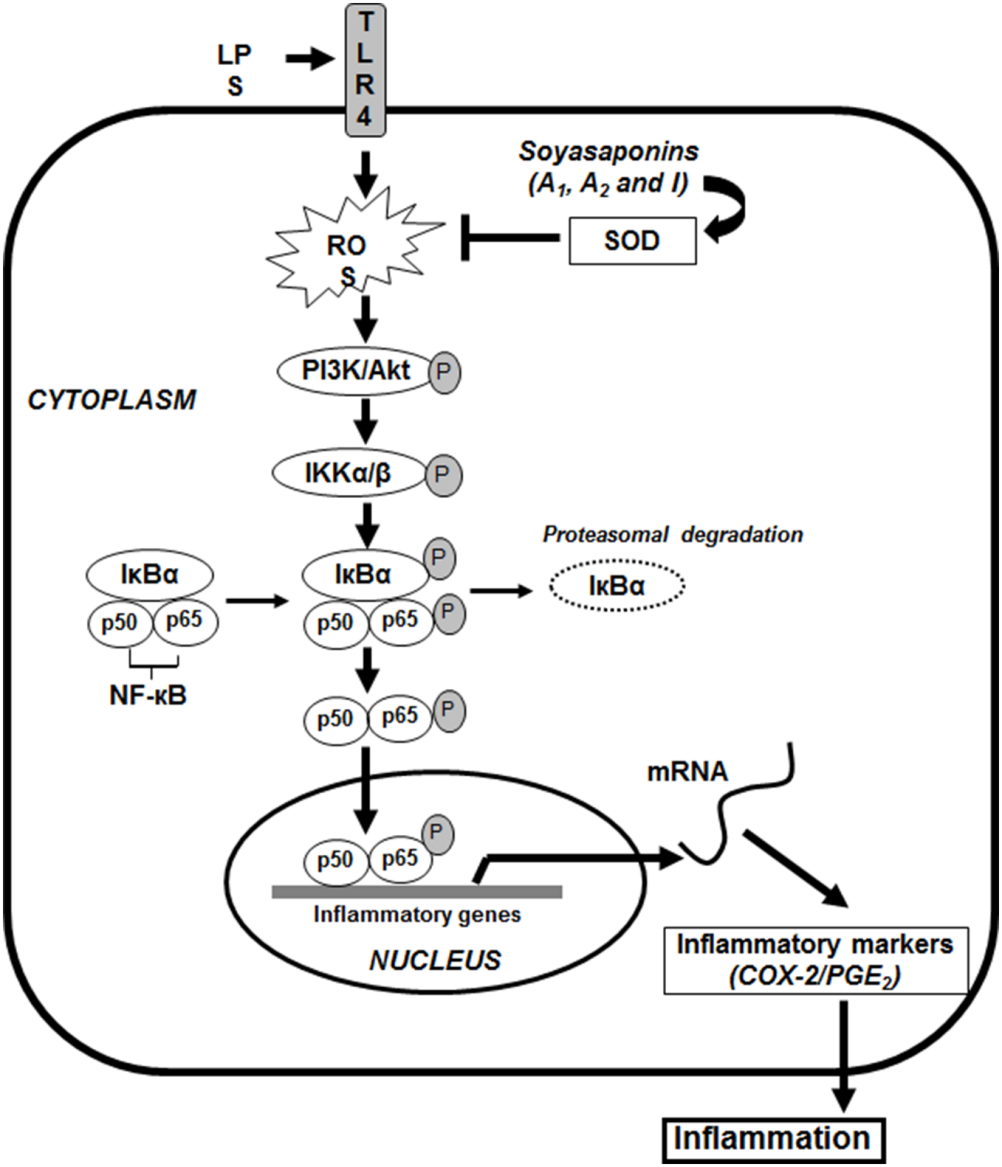
Schematic diagram of the targets of soyasaponins.

In summary, results from this study show that soyasaponins can affect activation of each step of the NF-kB signaling pathway. The main and new discoveries of this study are the effect of soyasaponins on the initiation steps of NF-kB signaling including the involvement of PI3K/Akt and SOD activation (**Fig. 9**). These results are important for explaining the multi-faucets beneficial effects of soyasaponins in human health.
